# Add-On Selective Estrogen Receptor Modulators for Methadone Maintenance Treatment

**DOI:** 10.3389/fendo.2021.638884

**Published:** 2021-08-05

**Authors:** Chieh-Liang Huang, Yao-Chang Chiang, Wei-Chun Chang, Yu-Ting Su, Juan-Cheng Yang, Wei-Chung Cheng, Hsien-Yuan Lane, Ing-Kang Ho, Wen-Lung Ma

**Affiliations:** ^1^Tsaotun Psychiatric Center, Ministry of Health and Welfare, Taichung, Taiwan; ^2^Sex Hormone Research Center, Center for Drug Abuse and Addiction, Chinese Medicine Research and Development Center, Department of Psychiatry, Department of OBS & GYN, China Medical University Hospital, Taichung, Taiwan; ^3^Department of Nursing, Division of Basic Medical Sciences, Chang Gung University of Science and Technology, Chiayi County, Taiwan; ^4^Graduate Institute of Biomedical Sciences, School of Medicine, China Medical University, Taichung, Taiwan; ^5^Department of Nursing, Asia University, Taichung, Taiwan

**Keywords:** methadone, sex disparity, SERM, opiate addiction, MMT

## Abstract

Methadone maintenance treatment (MMT) remains the cornerstone for the management of opiate abuse. However, MMT can be associated with complex factors, including complications during the tolerance phase, the inability of some patients to maintain treatment effects during the tapering or abstinence phases, and the development of methadone dependence. Previous studies have revealed a sex disparity in MMT efficacy, showing that women undergoing MMT experiencing an increase in psychological symptoms compared with men and suggesting a link between disparate responses and the effects of estrogen signaling on methadone metabolism. More specifically, estradiol levels are positively associated with MMT dosing, and the expression of a single-nucleotide polymorphism (SNP) associated with estrogen receptor (ER) regulation is also associated with MMT dosing. In addition to performing mechanistic dissections of estrogen signaling in the presence of methadone, past studies have also proposed the targeting of estrogen signaling during MMT. The present report provides an overview of the relevant literature regarding sex effects, including differences in sex hormones and their potential impacts on MMT regimens. Moreover, this article provides a pharmacological perspective on the targeting of estrogen signals through the use of selective ER modulators (SERMs) during MMT. Preliminary preclinical experiments were also performed to evaluate the potential effects of targeting estrogen signaling with tamoxifen on methadone metabolism.

## Introduction

The use of illicit opioids remains an ongoing issue for both individuals and society at large (1). Individuals who develop opioid dependency often experience personal or social problems and often engage in opioid abuse. Opioid abuse patients present with behavioral changes, such as social withdrawal (2); increased risk of contracting human immunodeficiency virus (HIV); and greater susceptibility to other opportunistic infections, such as hepatitis C and tuberculosis (TB) (3). In addition, individuals who abuse opiates exhibit higher rates of other infections (4); medical and psychiatric comorbidities (5); polysubstance consumption (5); and criminal behaviors, and an increase in opioid abuse has resulted in a surge in opioid-related deaths (6).

Methadone maintenance treatment (MMT) consists of the administration of methadone over a prolonged period of time to treat an individual addicted to opioids, such as heroin. MMT is typically used to treat individuals who experience relapse following typical detoxification regimens or admission to a substance abuse treatment facility that requires complete abstinence (7). MMT facilitates “social rehabilitation” by allowing people with addiction to avoid the uncomfortable withdrawal symptoms associated with complete abstinence. MMT has been used to treat opioid abuse patients for decades and has long served as the standard treatment for opiate dependence. However, MMT is associated with complications, including differential efficacies, and some patients experience difficulty during the transition from MMT to abstinence (8).

Appropriate dosing that aims to prevent the occurrence of withdrawal symptoms is a key component of the MMT strategy and is typically divided into three phases. When a patient first visits a treatment clinic, their craving for opiates is evaluated, and the initial dosage is determined and administered accordingly. The physician can then increase the dosage if the cravings are not mitigated by the initial dose. This stage is referred to as the “tolerance phase”, and during this period, the maximal methadone dose (MAX) is typically administered. Once a good balance between dosing and craving is achieved, the physician will gradually reduce the dosage to push the boundary at which the craving reoccurs. This stage of treatment is called the “tapering phase”, during which a steady-state (SS) methadone dosage is established. During this phase, social and mental support are typically provided to reduce the probability that the patient will seek the acquisition of street drugs. Finally, the physician will discontinue methadone dosing. This stage of MMT is called the “abstinence phase” and requires continued social and mental support. The evaluation of MMT regimen outcomes is based on the degree to which opiate-addicted patients can be successfully maintained in treatment programs, which contribute to the prevention of potential social impacts from crime and other addiction-related behaviors. However, significant numbers of opiate abusers who undergo MMT discontinue their treatment programs due to insufficient dosage, often in favor of the acquisition of street drugs (9, 10). Unfortunately, methadone overdose can be fatal (11), requiring strict constraints on dosing during the tolerance phase.

Studies published in recent years have reported sex-associated disparities in the efficacy and complication rates associated with MMT, although the results have varied across different geographic regions. Therefore, in the present report, we aimed to summarize the current knowledge regarding these issues and, through preliminary tests, provide important perspectives regarding potential treatment strategies for addressing them.

### Sex Disparities in MMT Efficacy

A sex disparity in the responses to MMT regimens has been reported, with women experiencing more psychological symptoms than men (12). In 2015, Bawor et al. (6) published a meta-analysis reviewing 22 clinical studies involving a total of almost ten thousand patients to evaluate the therapeutic outcomes of MMT. They found that men and women differed significantly in terms of alcohol use, amphetamine use, legal involvement, and employment while receiving MMT treatment. Sex differences were also prominent in terms of polysubstance use. Meanwhile, a national cohort study of ten thousand patients (the VEdeTTE study) also examined sex-related differences among MMT recipients (13). In that study, the women were younger and more likely to be married, divorced, or widowed than the men, and the women also had higher rates of unemployment despite being better educated on average. The men were reported to use sedatives more frequently and presented with higher rates of psychiatric comorbidities (such as depression, self-injury attempts, and suicide attempts). High doses of methadone and the use of methadone in combination with psychotherapy improved treatment retention for both men and women. Therefore, the VEdeTTE study results suggested that a sex-sensitive approach should be applied to improve MMT outcomes. Consistent with the VEdeTTE study, another national cohort study (14) also found that women receiving MMT had high rates of physical and psychological problems, including inherited psychiatric illnesses, and often began using opioids following a physician’s prescription. By contrast, a study by Leone et al. (12) found that MMT was significantly associated with higher psychological symptom scores in men compared with women. Wang et al. (15) reported that the levels of depression among women receiving MMT decreased faster than those in men receiving MMT.

When examining sex-specific differences in MMT outcomes, women are reported to have higher odds of discontinuing MMT to seek illicit opioids than men (16, 17). This discrepancy could be associated with pharmacodynamic differences because the methadone concentration-to-dose ratio (CDR) has been reported to be significantly lower among women than men (18). One study indicated that the MMT discontinuation rate could partially be attributed to MMT complications associated with prolonged QT intervals (19) (the QT interval is the time from the beginning of the QRS complex in an electrocardiograph, representing ventricular depolarization, to the end of the T wave, resulting from ventricular repolarization), although the results of another study did not support this theory (20). However, prolonged QT intervals could be associated with differences in the serum levels of sex hormones (21). Yee et al. (21) reported that men receiving MMT had testosterone levels lower than the reference range for male patients not receiving MMT. Interestingly, altered testosterone levels appear to be unrelated to prolactin levels (21), which suggests the involvement of steroidogenic cytochrome enzymes in the associated process. Kringen et al. (22) reported that the feminizing cytochrome gene family CYP2B6, which is regulated by estrogen signals, could be involved in regulating the CDR in MMT patients.

### The Impacts of Estrogen Signaling on MMT Efficacy

As early as the 1970s, a report described the complication of amenorrhea among women treated with methadone, which was associated with the secretion of gonadotropins from the hypothalamus (23). Therefore, methadone metabolism was suspected to be related to sex hormone production. For example, studies showed that the placental aromatase CYP19 mediated methadone metabolism and that methadone suppressed the aromatization of testosterone to estrogen (24, 25). The suppression of estrogen production might represent a long-term effect of methadone rather than an acute response (26). These studies strongly indicated that MMT could alter sex hormone production through CYP19 enzymes. In 2010, Lu et al. (27) discovered direct evidence showing that methadone is metabolized by CYP19 and may inhibit CYP19 *in vivo*.

Sex-related differences in methadone metabolites are prominent in the literature. Chalabianloo et al. (18) found that the CDR was significantly reduced among women compared with men, particularly in those women concurrently using CYP inducers and high doses of CYP3A4 inhibitors. Several lines of evidence suggest that CYP2B6, one of the feminizing CYPs, increases the drug clearance of methadone in pregnant women, and pregnancy is characterized by elevated estradiol (E2) levels (28). In addition, CYP2B6 can be activated by xenotropic agents, including the synthetic opioids pethidine and methadone, through the activation of the constitutive androstane receptor and pregnane X receptor in the liver (29).

In a recent study, Chiang et al. (30) showed that high estrogen levels were correlated with high MMT doses. Chiang et al. also showed that the expression of the ER-regulating CYP2B6 single-nucleotide polymorphism (SNP) was associated with MMT dosing. They replicated the phenotype observed in mice and found that the ablation of estrogen levels by ovariectomy in female mice suppressed methadone metabolism. By contrast, the implantation of E2 in male mice facilitated methadone metabolism. The manipulation of E2 levels also altered the addictive behaviors among mice addicted to methadone. The conditioned place preference (CPP) test, which measures retention time to evaluate the opioid craving status, was used to demonstrate that increased estrogen levels increased the retention time of methadone in both sexes.

Due to the existing complications associated with MMT, sex-related differences in efficacy, and interference of CYP19 in MMT efficacy, Chiang et al. proposed the potential of targeting estrogen receptor (ER) signaling to improve MMT efficacy. Therefore, we aimed to re-examine the effects of estrogen-ER signaling in MMT.

## Methodology

### Study Subjects

All experimental procedures for human studies were approved by the China Medical University Hospital (CMUH) Institutional review board (DMR94-IRB-007), and informed consent was obtained from each subject. The cohort consisted of 326 heroin abusers (age range: 20–70 years) recruited from January 2010 – December 2013 from among psychiatric outpatients treated by CMUH in Taichung, Taiwan. In addition, each patient received methadone therapy for at least 6 months and maintained an unchanged methadone dosage for at least 4 weeks prior to recruitment. Subjects who received other medications that might affect methadone metabolism or who had any Diagnostic and Statistical Manual of Mental Disorders, fourth edition (DSM-IV) Axis I or II psychiatric disorders were excluded from this study. At the time of enrollment, all subjects were asked to complete a questionnaire that included demographic data and a survey of heroin addictive behavior.

### Generation and Housing of ERα and ERβ Knockout Mice

All animal experiments were performed in accordance with the Guide for the Care and Use of Laboratory Animals of the Ministry of Science and Technology and were conducted with approval from China Medical University (approval number #103-36-N). The ERα-knockout (KO) mice (*ActbCre-ERα^loxP/loxP^*) and ERβ-KO mice (*ERβ^–/–^*) used in our study were kindly provided by Prof. Shuyuan Yeh and Prof. Chawnshang Chang, respectively, at the University of Rochester, NY, USA (31, 32). To generate ERα-KO mice (33, 34), transgenic *ERα^loxP/loxP^* mice were crossed with *ActbCre* (β-actin promoter–driven Cre recombinase) transgenic mice to generate male ERα-KO (*ERα^–/–^*) mice. The control mice were *ERα^loxP/loxP^* without *ActbCre*. ERβ-KO (*ERβ^–/–^*) mice were generated by crossing heterozygote (*ERβ^+/–^*) mice and littermate wild-type (*ERβ^+/+^*) mice. PCR was used to identify the mouse genotypes from DNA obtained from tail skin treated overnight with cell lysis buffer containing 0.5 mg/ml proteinase K (Sigma, P2308). All wild-type *vs. ERα^–/–^* or *ERβ^–/–^* mice used in these studies were 2–4 months old and male. All protocols related to animal use and treatment were evaluated and approved by the Animal Care and Use Committee of China Medical University, and all animals were treated in accordance with National Laboratory for Experimental Animals guidelines.

### E2/Tamoxifen Injection Protocol

The E2/tamoxifen (TMX) injection procedure was performed as previously described (35). In brief, E2 (Sigma-Aldrich) or TMX (Sigma-Aldrich) were dissolved in sesame oil:ethanol vehicle (9:1, v:v). Each mouse was injected subcutaneously (s.c.) with either 0.01 mL vehicle or vehicle containing 20 µg E2 or 100 µg TMX for 4 consecutive weeks, 3 times/week.

### Tail-Flick Assay

The tail-flick test was performed in mice using a modified version of the method described by Dai et al. (36) The tail-flick latency was defined by the time (in seconds) for the animal to withdraw its tail from a heat source (bulb, 8 V/50 W, OSRAM, Germany), and was measured using a semi-automated machine (Columbus Instruments, Columbus, OH, USA). The infrared intensity of the tail-flick machine was set at 8, which produced a baseline tail-flick latency of 2–3 seconds, and the cutoff time was set to 10 seconds to prevent tissue damage. The mice were adapted to the restrainer for 5 min prior to performing the tail-flick test. To measure the analgesic effects of opioid agonists, the animals were subjected to the tail-flick procedure once per day to minimize learning effects. All of the experimental animals were randomly assigned from different cages to ensure a general effect in the population. The antinociceptive effects were presented as the area under the time-response curve (AUC = latency × time).

### SNP Variant Selection

All SNPs identified in methadone metabolism–related enzymes [opioid-related nociceptin receptor 1 (OPRL-1) and CYP2B6] were analyzed (using the UCSC genome browser; http://genome.ucsc.edu). The putative estrogen response element (ERE) areas were identified by comparing the results predicted by the TFSEARCH website (http://www.cbrc.jp/research/db/TFSEARCH.html) and the PReMod (37) database (http://genomequebec.mcgill.ca/PReMod/). The genotypes of ERE-SNP alignment scores were determined using TheBEST (The Binding Element Searching Tool; http://thebest.binfo.ncku.edu.tw/thebest/) algorithm.

### DNA Isolation and Genotyping

Genomic DNA was extracted from 8–10 ml peripheral whole-blood samples using the MasterPure™ DNA Purification Kit for Blood Version II (Epicentre, Madison, WI, USA). DNA specimens were dissolved in Tris-EDTA (TE) buffer and stored at −20°C until PCR. All of the ERE-SNPs (**Table 1**) were determined using Sequenom iPLEX MALDI-TOF (matrix-assisted laser desorption ionization-time of flight; Sequenom Inc., San Diego, CA, USA), according to the manufacturer’s protocol.

**Table 1:** Demograph of MMT cohort in association of SNP and MMT dosing.

		<50 mg (87 patients)	51~100 mg (163 patients)	>100 mg (45 patients)	
					p-value
**Sex**	Male	69 (29.4%)	133 (56.6%)	33 (14.0%)	0.3446
	Female	18 (29.5%)	30 (49.2%)	13 (21.3%)	
**Age**		41.0±7.3	41.9±7.4	38.3±7.0	0.0158
**BMI**		22.8±3.1	22.7±2.7	22.6±2.4	
**HIV**	HIV (-)	51/51 (100%)	150/154 (97.4%)	64/66 (96.97%)	0.9176
	HIV (+)	0/51 (0%)	5 (83.3%)	1 (16.7%)	
					*p*-value
**OPRL-1: rs7271530**	TT	21 (26.6%)	55 (36.7%)	13 (32.5%)	0.0747
	CC	14 (17.7%)	17 (11.3%)	11 (27.5%)	
	CT	44 (55.7%)	78 (52.0%)	16 (40.0%)	
** Dominant**	**TT+CT**	**65 (82.3%)**	**133 (88.7%)**	**29(72.5%)**	**0.0361**
	**CC**	**14 (17.7%)**	**17 (11.3%)**	**11(27.5%)**	
Recessive	CT+CC	58 (73.4%)	95 (63.3%)	27 (67.5%)	0.3036
	TT	21 (26.6%)	55 (36.7%)	13 (32.5%)	
**OPRL-1: rs6010717**	GG	29 (37.2%)	70 (46.7%)	20 (51.3%)	0.1413
	CC	12 (15.4%)	9 (6.0%)	3 (7.7%)	
	CG	37 (47.4%)	71 (47.3%)	16 (41.0%)	
** Dominant**	**GG+CG**	**66 (84.6%)**	**141 (94.0%)**	**36(92.3%)**	**0.0361**
	**CC**	**14 (17.7%)**	**17 (11.3%)**	**11(27.5%)**	
Recessive	CG+CC	49 (62.8%)	80 (53.3%)	19 (48.7%)	0.3036
	GG	29 (37.2%)	70 (46.7%)	20 (51.3%)	
**OPRL: rs2229205**	CC	59 (74.7%)	111 (73.0%)	27 (67.5%)	0.1991
	TT	0 (0.0%)	4 (2.6%)	3 (7.5%)	
	CT	20 (25.3%)	37 (24.4%)	10 (25.0%)	
Dominant	CC+CT	79 (100%)	148 (97.4%)	37 (92.5%)	0.0513
	TT	0 (0.0%)	4 (2.6%)	3 (7.5%)	
Recessive	CT+TT	20 (25.3%)	41 (27.0%)	13 (32.5%)	0.7013
	CC	59 (74.7%)	111 (73.0%)	27 (67.5%)	
**CYP2B6: rs16974799**	**CC**	**41 (51.9%)**	**101 (66.5%)**	**31(77.5%)**	**0.0374**
	**TT**	**33 (41.8%)**	**4 (2.6%)**	**0(0.0%)**	
	**CT**	**5 (6.3%)**	**47 (30.9%)**	**9(22.5%)**	
Dominant	CC+CT	74 (93.7%)	148 (97.4%)	40 (100%)	**0.1477**
	TT	5 (6.3%)	4 (2.6%)	0 (0.0%)	
** Recessive**	**CT+TT**	**38 (48.1%)**	**51 (33.6%)**	**9(22.5%)**	**0.0138**
	**CC**	**41 (51.9%)**	**101 (66.5%)**	**31(77.5%)**	

Bold value indicating a significance difference between groups.

### Chemicals, Reagents, and Cell Culture

Methadone hydrochloride (USP, USA) was dissolved in distilled water and administered s.c. in a volume of 1.0 ml/kg body weight. E2 and TMX were obtained from Sigma-Aldrich (CA, USA).

### Enantiomeric EDDP/Methadone Detection by LC–MS/MS

The sample preparation and measurement of the R/S-forms of enantiomeric methadone or 2-ethylidene-1,5-dimethyl-3,3-diphenylpyrrolidine (EDDP), a methadone metabolite, were performed as previously described, with modifications (38, 39). In brief, each sample was prepared as follows: standard (R, S)–methadone or EDDP was purchased from Sigma (Sigma-Aldrich, CA, USA). For (R,S)–methadone and EDDP, the calibration curve points were: 0, 100, 250, 500, 1000, and 2000 ng/ml. Liquid chromatography-tandem mass spectrometry (LC–MS/MS) analysis was performed on an API 2000 LC–MS/MS system (AB Sciex, Ontario, Canada), interfaced with a high-performance liquid chromatography (HPLC) pump equipped with an autosampler (1100 series, Agilent, Waldbronn, Germany). A 50 µl volume of plasma sample was mixed with 100 µl of internal standard (EDDP-D3) and filtered for use. After 2 minutes of vortexing, the sample was centrifuged at 15,000 x *g* for 15 min, and the supernatant was applied for LC–MS/MS analysis. A Chiralcel OD-R column (250 x 4.6 mm, 5-µm particle size, Daicel Chemical Industries Ltd., Japan) was used, and the isocratic mobile phase system was run under a flow rate of 0.5 mL/min (phase A: 0.1% formic acid in acetonitrile, phase B: 10 mM ammonium acetate). The Q1/Q3 of EDDP was 278.3/234.3, whereas that of methadone was 310.1/265.6, and that of EDDP-D9 was 319.3/268.3.

### E2 Detection by ECLIA Assay

Electrochemiluminescent immunoassay (ECLIA) was used for the quantitative determination of estrogen levels in mouse serum on a Roche Elecsys 2010 instrument (Roche, Basel, Switzerland), according to the manufacturer’s instructions. The chemiluminescence reaction for the detection of the reaction complex was initiated by applying a voltage to the sample solution, resulting in a precisely controlled reaction. Serum E2 values are provided as pg/ml (pg/ml × 3.67 = pmol/l). The functional sensitivity of the E2 assay was 5 pg/ml (18.4 pmol/l), with a total analytical sensitivity of <5%.

### Statistical Analysis

Student’s t-test was used to assess differences between luciferase activity across genotypes. In addition, we also employed Student’s t-test to compare the different methadone doses and sex hormone levels in patients between sexes during the trials. Correlations were analyzed between sex hormone levels and the EDDP/methadone ratios. The pharmacokinetic data were analyzed with a two-way analysis of variance (ANOVA). The significance level was set to a two-sided P < 0.05. All statistical analyses were performed using SAS version 9.4 (SAS Institute, Inc, Cary, North Carolina, USA).

## Result

### Estrogen Receptors Directly Modulate Methadone Metabolism to Alter MMT Efficacy

**Figure 1:**
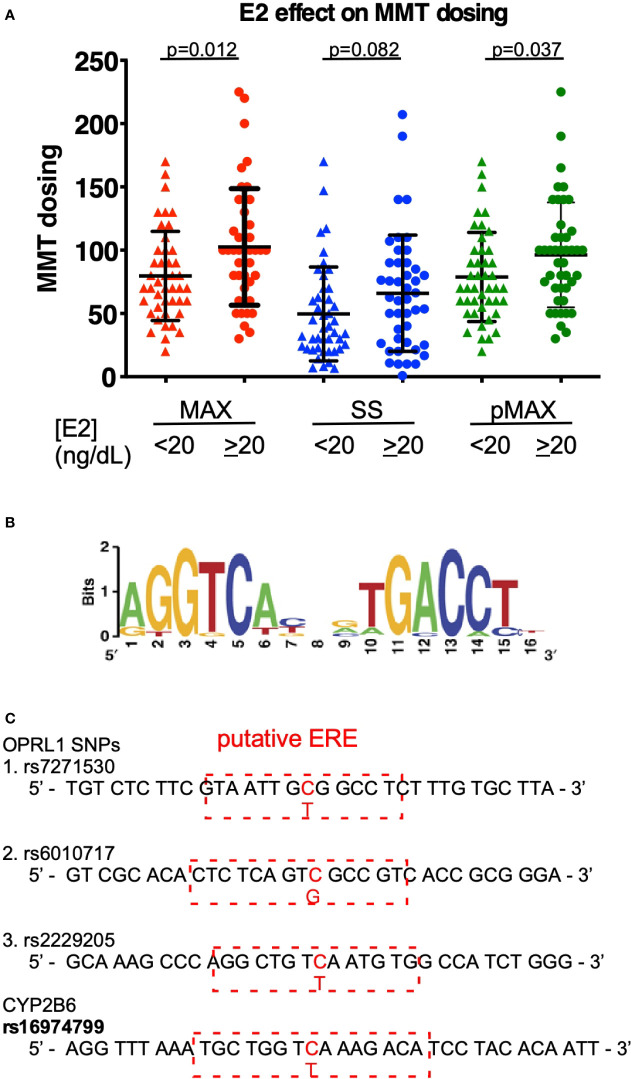
E2 effects on MMT dosing. **(A)** The MMT dosing in patients with low (<20 ng/dL) or high (≥20 ng/dL) estradiol (E2) levels. MAX (red-colored dots) indicates the maximal dosing of methadone during the MMT regimen; SS (blue-colored dots) indicates the steady-state dosing of methadone during the MMT regimen; and pMAX (green-colored dots) indicates the previous MAX of patients receiving a previous term of MMT. **(B)** The classical ERE sequences and scoring standard for predicting a potential SNP-ERE. **(C)** The locations of the four SNPs on the putative ERE. The *OPRL1* SNPs (rs7271530, rs6010717, and rs2229205) and *CYP2B6* SNP (rs1694799) sequences are shown, and red-dashed rectangles depict the putative ERE region and nearby sequences. The * indicates a significant p-value < 0.05 obtained using Student’s t-test.

To correlate estrogen levels with the outcomes of MMT, we measured the E2 levels of patients undergoing an MMT program in a cohort study. As shown in **Table 1**, sex, BMI, and HIV infection status appeared to have little effect on the MMT dosing level among the cohort, although age did have an effect, with younger ages correlated with higher MAX MMT dosages. We also measured the E2 levels of the patients in this cohort and found that they were associated with the MAX dose during the tolerance phase, the SS dose at the onset of the tapering phase, and the previous term of MAX dosing (pMAX). As shown in **Figure 1A**, higher E2 levels (cutoff 20 ng/dL) were associated with higher MAX and pMAX values.

Using the well-known ERE (**Figure 1B**), SNP sequence alignments were performed to determine which genes are correlated with the opioid response and opioid metabolism. We identified four SNPs with high prediction scores, including the three SNPs in *OPRL1* (rs7271530, rs6010717, and rs2229205) and one SNP in *CYP2B6* (rs16974799; **Figure 1C**). As shown in **Table 1**, these SNPs were preferentially correlated with the MMT MAX value. Among the *OPRL1* SNPs, rs7271530 (T-variant; dominant inheritance) and rs6010717 (G-variant; dominant inheritance) were associated with high MMT MAX values. In addition, the *CYP2B6* SNP rs16974799 (C-variant; recessive inheritance) was also associated with a high MMT MAX value. These data indicated the potential for the direct regulation of estrogen/ER signaling to affect the MMT response.

**Figure 2:**
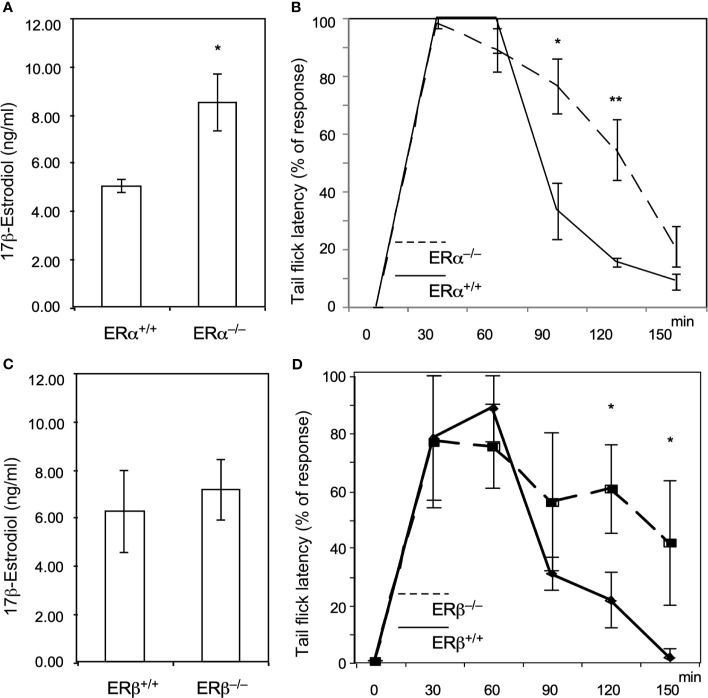
Knock out of ERα and ERβ in male mice resulted in prolonged analgesic responses to methadone treatment. **(A)** 17β-estradiol levels (ng/dL) in wild-type (ERα^+/+^; n = 6; solid line) or ERαKO (ERα^−/−^, n = 6; dashed line) mice. **(B)** Tail-flick latency tests in wild-type and ERαKO mice. The time at which each mouse’s tail flicked (in seconds) was measured 30, 60, 90, 120, and 150 min after methadone injection (*s.c.*; 1.0 ml/kg body weight). **(C)** 17β-estradiol levels (ng/dL) in wild-type (ERβ ^+/+^; n=6) or ERβKO (ERβ^−/−^, n = 6) mice. **(D)** Tail-flick latency tests in wild-type (solid line) and ERβKO (dashed line) mice. The time at which each mouse’s tail flicked (in seconds) was measured 30, 60, 90, 120, and 150 min after methadone was injected (*s.c.*; 1.0 ml/kg body weight). Each group of data were collected from 6 mice. The * indicates a significant p-value < 0.05, and ** indicates p-value < 0.01, obtained using Student’s t-test.

To verify that ERs (ERα and ERβ) are involved in the methadone response, we used ERα and ERβ KO mice to test the acute effects of methadone. As shown in **Figure 2A**, ERα KO increased E2 levels, and the tail-flick test showed a prolonged analgesic response to methadone in ERα KO mice compared with wild-type mice (**Figure 2B**). By contrast, the ERβ KO mice did not exhibit altered E2 levels (**Figure 2C**), although they also exhibited a prolonged analgesic response to methadone compared with wild-type mice (**Figure 2D**). These data provide direct evidence that estrogen signaling decreased the methadone response through both ERα and ERβ in mice.

### The Potential Strategies for Targeting Estrogen Signaling in MMT

Two major estrogenic ablation therapies are currently used to treat estrogen-sensitive breast cancer. One consists of inhibiting the ER function using with SERMs, whereas the other consists of reducing estrogen production through the use of aromatase inhibitors (AIs) to inhibit enzymatic activity. The following section will discuss the potential use of anti-estrogenic agents, such as SERMs or AIs, as an add-on therapy during MMT.

The pharmacological action of AIs involves the inhibition of aromatase enzymatic activity to suppress estrogen signaling by inhibiting E2 production in the ovary/liver (40) and adipose tissues (41). AIs have been demonstrated to influence female reproductive systems. For example, Wu et al. (42) conducted a randomized trial using the AI anastrozole and found that it was associated with high pregnancy rates, although treatment was also associated with fewer ovulatory follicles compared with treatment by clomiphene citrate. In another study, Tabatabaie et al. (43) used the AI letrozole combined with medroxyprogesterone acetate to treat endometrial hyperplasia and found that it exhibited good performance for preserving fertility in young women. In a recent study, AIs were shown to be useful for ovarian stimulation prior to oocyte/embryo cryopreservation in estrogen-sensitive cancer patients (44).

The pharmacological action of SERMs involves the suppression of estrogenic signaling through the inhibition of ER function by preventing the estrogen-induced transactivation of ERs. However, the use of the SERM TMX in female patients has also been associated with complications. For example, as early as 1998, Mishell et al. (45) reported that TMX administration followed by intravaginal misoprostol could be used to induce abortion in pregnancies as late as 56 days of gestational age. SERMs, including clomiphene citrate and TMX, have also been used as the first-line treatment of choice for anovulation (46). Shandley et al. (47) and Wright et al. (48) also reported that women treated with TMX were more likely to experience gynecological complications than those treated with AIs.

From these findings, AIs might appear to be a better choice for use with MMT regimens. However, the effects of these add-ons in male patients, which constitute the majority of MMT patients, must also be considered. In addition, a recent report indicated that AIs could increase the chances of developing insulin insensitivity (49), which represents another potential factor that should be considered prior to using AIs in MMT. Some reports have suggested that the use of AIs in male patients could lead to increased sexual activity and increased erectile ability (50, 51). In addition, the use of AIs could also increase the chances of pregnancy in females, compared with the use of SERMs (43).

Chiang et al. (30) tested estrogen signal targeting using SERMs in MMT by adding the SERM TTX to the tolerance, tapering, and abstinence phases of MMT in a mouse model. The results showed excellent tolerance behavior, and the addition of TMX to MMT also enhanced the CPP retention time, indicating that TMX has the potential to relieve stress-induced cravings for opioids during the tolerance phase of MMT programs, which could prevent the chances of a methadone overdose. More strikingly, the addition of TMX to the tapering and abstinence phases rapidly reduced the CPP retention time, which indicates that the addition of TMX to the MMT protocol was able to reduce drug-seeking behavior even as the methadone dose was reduced.

**Figure 3:**
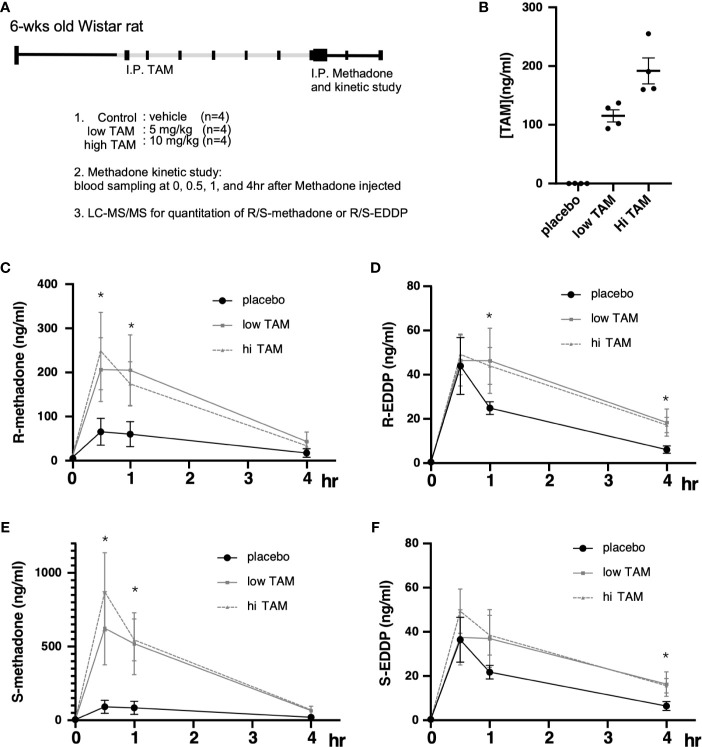
Pharmacodynamic alterations in methadone metabolism with or without tamoxifen (TAM) injections. **(A)** Experimental design and treatment procedures. The 6-wk-old Wistar rats were randomly assigned to each of the study groups. First, TAM injections (control; low, 5 mg/kg; or high, 10 mg/kg) were injected for 6 days, followed by 10 mg/kg methadone on day 7, and blood was drawn at 0, 1, 2, 3, and 4 h after methadone injection to measure R/S-methadone, R/S-EDDP, and TAM using LC–MS/MS. **(B)** The serum TAM levels were measured at 4 h after methadone injection. **(C)** The serum R-methadone levels were measured in each group. **(D)** The serum R-EDDP levels were measured in each group. **(E)** The serum S-methadone levels were measured in each group. **(F)** The serum S-EDDP levels were measured in each group. The **** indicates a significant p-value < 0.00001, obtained using two-way ANOVA to compare groups. *I.P: intra-peritoneal injection. The * indicates a significant p-value < 0.05, obtained using two-way ANOVA to compare groups. I.P., intra-peritoneal injection.

Although the study by Chiang et al. showed that one phenotype observed in response to targeting ERs with TMX was beneficial for MMT program efficacy, whether TMX exerts pharmacological effects on methadone metabolism remains unclear. Therefore, we conducted a longer-term treatment consisting of methadone with and without TMX to observe the levels of racemic methadone (R/S-methadone) and its metabolite R/S-EDDP (2-ethylidene-1, 5-dimethyl-3, 3-diphenylpyrrolidin). We used a rat model that was injected with TMX for 6 days, followed by methadone injection on day 7, and the methadone/EDDP concentrations in the serum were determined (**Figure 3A**). Different groups of animals were injected with placebo, low (5 mg/kg), and high (10 mg/kg) doses TMX, after which the TMX concentration in the serum was measured. The results obtained showed that the serum concentrations of TMX were differentially elevated in response to the various injection doses (**Figure 3B**). The three groups of rats were intraperitoneally injected with methadone, and racemic R/S-methadone and its metabolite R/S-EDDP were monitored for 4 h. As shown in **Figure 3C**, the serum R-methadone retention times were prolonged, and the Cmax (maximal/peak serum concentration) values were significantly increased. For R-EDDP (**Figure 3D**), the retention time was prolonged, but the Cmax values for R-EDDP remained unchanged. By contrast, the S-methadone retention times were prolonged, and the associated Cmax values were dramatically increased (**Figures 3E, F**). In addition, the S-EDDP retention time was prolonged, but the Cmax values of S-EDDP remained unchanged. R-methadone is the pharmacologically active racemate, whereas S-methadone is the racemate associated with complications (52, 53); therefore, TMX may not be the ideal SERM for use as an add-on to MMT. The mechanism underlying the superior suppressive effect on S-methadone metabolism may be associated with the TMX-mediated inhibition of CYP2B6 being more effective in experimental rodents compared with the inhibition of human CYP2C19 (30). However, the ability to utilize TMX to suppress ER function and prolong methadone metabolism was confirmed by this study.

**Figure 4:**
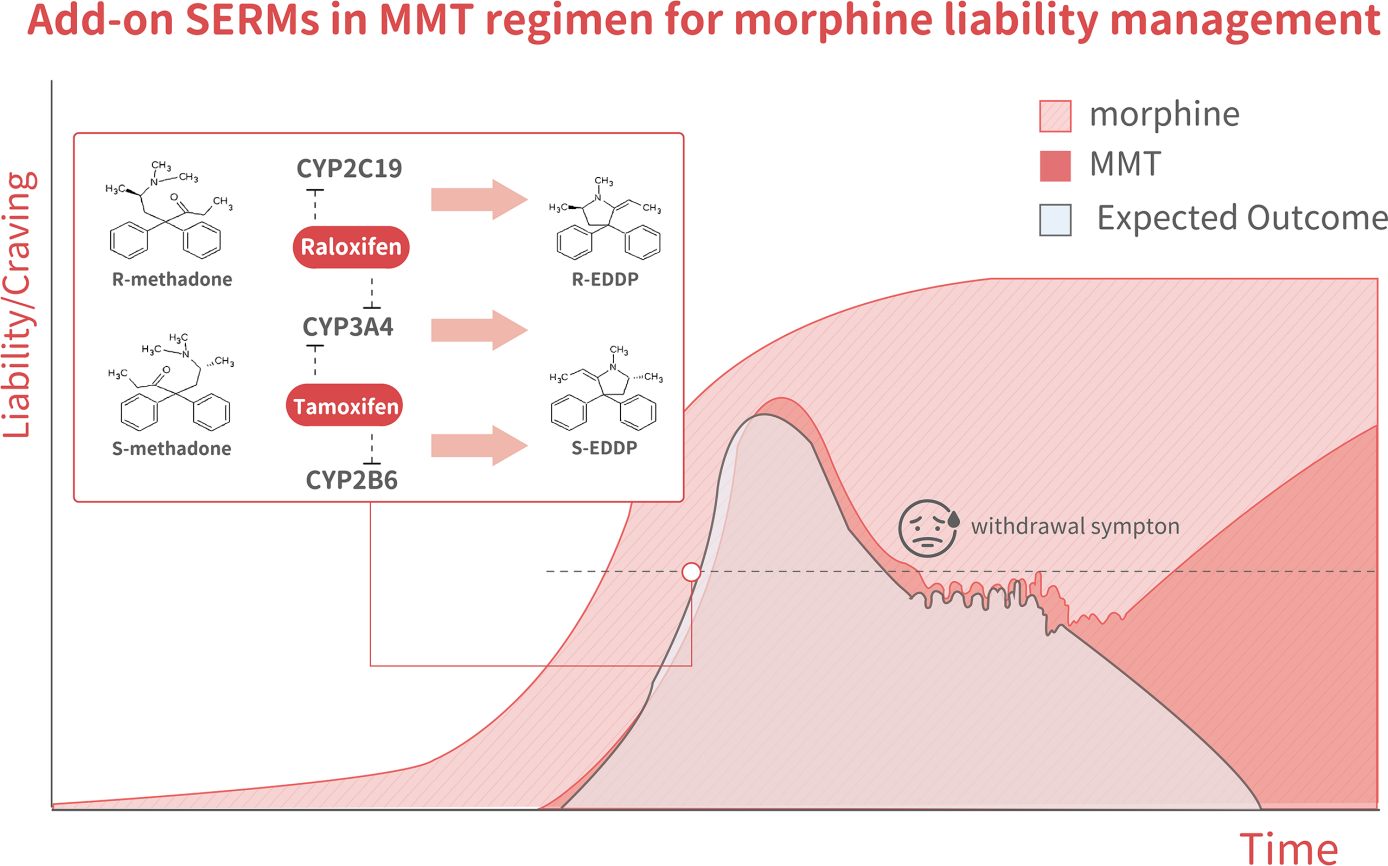
The model illustrates changes in methadone liability and cravings during MMT, which is associated with three outcome scenarios. The dark-pink area shows the MMT regimen-related liability to quickly increase over the tolerance phase, decline over the taper phase, but then increase again during the abstinence phase. The light pink area indicates morphine-related liability. The light blue areashows the expected outcome of methadone + SERMs, with a liability to increase but to a lesser degree than in mice receiving methadone alone during the tolerance phase. It declined to an even lower degree during both the tapering and abstinence phases. The inset shows a schematic illustration of methadone metabolism. Racemate catalyzation is selectively affected by various SERMs. R-methadone is primarily catalyzed by CYP2C19, whereas CYP2B6 catalyzes S-methadone. CYP3A4 can catalyze both racemates. Tamoxifen was found to suppress CYP2B6 predominantly through the inhibition of gene expression and the suppression of CYP3A4 through direct interaction.

In methadone metabolism, S-racemate is majorly metabolized by CYP2B6, whereas the R-racemate is metabolized by CYP2C19 (54, 55). In addition, CYP3A4 is a potent methadone-metabolizing enzyme without an indicated preference for either racemate (56). Several clinically available SERMs can be tested as add-on anti-ER agents in MMT. Raloxifene is a SERMs that has been reported to suppress CYP3A4 and CYP2C19 (57). Raloxifene has been reported to inactivate CYP3A4 kinetics with K(I) and k(inact) values of 0.81 µM and 0.20 min^−1^, respectively (57), by forming a homodimer within the CYP3A4 protein (58, 59). Other SERMs, such as ospemifene (60, 61) (a non-steroidal SERM) and LY2066948 (62) (which was developed by Eli Lilly to treat uterine fibroid myomas), could be also be considered for add-on MMT therapy given their excellent interactions with CYP3A4 and CYP2C19. Therefore, raloxifene, ospemifene, and LY2066948 could potentially be tested as add-on agents in MMT for opiate abuse patients. As indicated in **Figure 4**, the selective antagonism of R/S-methadone racemates that can be achieved by suppressing CYP2C19, CYP2B6, or CYP3A4 with SERMs might represent an excellent strategy for the future development of new MMT regimens.

## Conclusion

In this perspective article, we reviewed the roles of sex disparities in MMT recipients and tested the possibility of using a SERM, specifically TMX, to affect methadone metabolism. Our report indicated that the addition of an effective SERM to MMT regimens might exert beneficial effects by extending methadone metabolism, as the addition of TMX increased the Cmax in a rat model. Although the use of TMX led to more robust increases in S-methadone, which is associated with complications, future testing with other SERMs would be of great interest.

## Data Availability Statement

The raw data supporting the conclusions of this article will be made available by the authors, without undue reservation.

## Ethics Statement

All of the experimental procedures of the human studies were approved by the China Medical University Hospital (CMUH) Institutional review board (DMR94-IRB-007), and informed consent was obtained from each subject. The patients/participants provided their written informed consent to participate in this study. All protocols related to animal use and treatment were evaluated and approved by the Animal Care and Use Committee of China Medical University, and all animals were treated in accordance with National Laboratory for Experimental Animals guidelines.

## Author Contributions

C-LH and W-CCha recruited patients, performed statistical analyses, and composed the manuscript. Y-CC and J-CY executed the animal experiments, performed statistical analyses, and edited the manuscript. Y-TS assisted with the animal experiment, LC–MS/MS, and molecular biology studies. Grants to H-YL and I-KH supported this study, and they participated in associated discussions and edited the manuscript. W-LM initiated the study, interpreted the data, supported the entire project, and edited and approved the final manuscript. All authors contributed to the article and approved the submitted version.

## Funding

Financial support: Taiwan Ministry of Science and Technology [MOST109-2320-B-039-006; MOST108-2314-B-039-043-MY3], Taiwan National Health Research Institute [H-YL, NHRI-EX108-1073NI; W-LM, NHRI-EX109-10705BI], and CMU/CMUH [CMU108-MF-33, DMR-108-080, DMR-109-084, and DMR-109-240].

## Conflict of Interest

The authors declare that the research was conducted in the absence of any commercial or financial relationships that could be construed as a potential conflict of interest.

## Publisher’s Note

All claims expressed in this article are solely those of the authors and do not necessarily represent those of their affiliated organizations, or those of the publisher, the editors and the reviewers. Any product that may be evaluated in this article, or claim that may be made by its manufacturer, is not guaranteed or endorsed by the publisher.
